# Editorial Comment: Quality of life in enuretic children

**DOI:** 10.1590/S1677-5538.IBJU.2020.0308.1

**Published:** 2021-03-25

**Authors:** Lisieux Eyer de Jesus

**Affiliations:** 1 Universidade Federal Fluminense Hospital Universitário Antônio Pedro NiteróiRJ Brasil Serviço de Urologia Pediátrica, Hospital Universitário Antônio Pedro - Universidade Federal Fluminense - UFF, Niterói, RJ, Brasil

## COMMENT

Congratulations to the authors, not only for their excellent research, but also for their widely recognized dedication to the treatment of pediatric voiding dysfunction, which made them able to establish a high quality public specialized medical servisse (1).

To evaluate quality of life in childhood is difficult and depends on various aspects, influenced by culture and social environment. Medical conditions that disproportionately affect specific aspects of life add a layer of complexity to employ “general” questionnaires: some diseases may harm children deeply, despite showing no effects over many of the variables studied. I suspect that enuresis is one of those illnesses.

Enuresis socio-psychological consequences are universal, but deeply influenced by cultural and social aspects. For example, in cultures where sleep-overs are a common form of socialization for school-aged children, such as in the USA, not being able to participate in such activities are seriously limiting.

In Brazil, house conditions and ignorance impact on the families´ attitudes towards enuresis. In our poor counties, it is common for children to share their beds and bedrooms with other people, including adults. In those circumstances, complaints, disgust, and even swearing towards children are common.

Also, illiterate parents have difficulties to understand enuresis as a disease, and tend to interpret the problem as behavioral. Children are frequently punished or publically shamed after enuresis episodes, “for them to learn”.

Even the non-availability of household facilities influence the tolerance of the families towards enuresis: a family I used to treat changed their behavior towards the child from aggressive to tolerant after saving enough money to buy a washing machine….

A practical aspect to consider is the difficulties to obtain governmental funding to treat enuretic children. Desmopressin and enuresis alarms are relatively expensive: each cost approximately 40 American dollars, and in our country minimum wages are circa 200 American dollars. Their price make treatment impossible for a lot of poor children.

This research demonstrated the socio-psychological negative consequences of enuresis in children. Its results may facilitate our public services to prove the need for psychologists and social workers in the multidisciplinary team dedicated to treating Pediatric Voiding dysfunction (1). Also, it documents that paying for enuresis treatment, despite addressing a disease that does not imply death or disability, is justified to treat or impede serious familiar and socio-psychological permanent problems.
